# Childhood maltreatment and suicidal ideation in Chinese children and adolescents: the mediating role of mindfulness

**DOI:** 10.1186/s12888-022-04336-w

**Published:** 2022-11-04

**Authors:** Xuemeng Liang, Wei Chang, Hailiang Ran, Die Fang, Yusan Che, Sifan Wang, Lin Chen, Hao Sun, Jin Lu, Yuanyuan Xiao

**Affiliations:** 1 Department of Epidemiology and Health Statistics, School of Public Health, Kunming Medical University, Kunming, China; 2 Psychiatry department, The First Affiliated Hospital, Kunming Medical University, Kunming, China; 3 Mental Health Institute of Yunnan, The First Affiliated Hospital, Kunming Medical University, Kunming, Yunnan China; 4 Yunnan Clinical Research Center for Mental Health, Kunming, Yunnan China

**Keywords:** Childhood maltreatment, Suicidal ideation, Mindfulness, Children and adolescents, Mediation

## Abstract

**Background:**

Childhood maltreatment (CM) has been associated with suicidal ideation (SI) in children and adolescents, yet the mediating role of mindfulness in this association remains unclear. This study aims to test the mediation of mindfulness in CM-SI association among a large sample of Chinese children and adolescents.

**Methods:**

A population-based cross-sectional study of 3455 children and adolescents aged 10–17 years from southwest China Yunnan province was conducted. Information from the participants was collected by using a comprehensive self-administered questionnaire. The Chinese version of the Childhood Trauma Questionnaire (CTQ), Five Facet Mindfulness Questionnaire (FFMQ), and the Beck Scale for Suicidal Ideation (BSSI) were used to measure CM, mindfulness and SI. Univariate and multivariate binary logistic regression models were used to estimate the crude and adjusted associations between CM, mindfulness and SI (one-week, one-year, lifetime). Pathway analysis was subsequently performed to test the mediation of mindfulness in CM-SI association.

**Results:**

The findings showed that mindfulness, CM and SI were significantly correlated with each other (*p* < 0.05). Mindfulness is a significant mediator in CM-SI association, accounted for 7.5, 11.4, and 17.6% of the total associations for one-week SI, one-year SI, and lifetime SI, respectively. For the five types of CM, the highest level of mediation via mindfulness had been found for physical neglect (PN) and one-year SI (34.3%), followed by emotional neglect (EN) and one-year SI (30.2%), sexual abuse (SA) and one-year SI (25.4%).

**Conclusions:**

Our study findings highlight the intervention potential of mindfulness in preventing CM associated suicidal risk. Future longitudinal studies are warranted to corroborate the effectiveness of mindfulness-based intervention for Chinese children and adolescents.

**Supplementary Information:**

The online version contains supplementary material available at 10.1186/s12888-022-04336-w.

## Background

In recent years, youth suicide is keep increasing at a significant rate and it is currently the second leading cause of death for children and adolescents [1]. Suicide is in essence a continuum, it starts from suicidal ideation (SI), via suicidal plan, suicidal attempt, ends at completed suicide [2]. From this perspective, proactively searching for associated factors of SI could be effective in preventing subsequent suicidal behaviors. In children and adolescents, identified influential factors of SI include demographics (age, sex, educational level, etc.), mental disorders (depression, anxiety, bipolar disorder, etc.), interpersonal difficulties and family conflict [3, 4].

Childhood maltreatment (CM) refers to the abuse or neglect that occur to children under 16 years of age [5]. The prevalence of CM is high: it is reported that nearly 3 in 4 children aged 2–4 years are suffering from physical punishment and/or psychological violence by their parents or caregivers [6]. CM imposes serious and profound psychological impairment to the victims: compared to non-abused counterparts, children and adolescents who had experienced CM showed higher level of generalized guilt, shame, depression, and anxiety [7, 8]. CM also relates to increased risk of SI: findings from the National Comorbidity Survey Replication (NCS-R) suggested that CM was associated with an increased odds of SI (OR = 1.80) in 5665 US adults [9]; in another newly published cross-sectional study of 3146 Chinese children and adolescents, study subjects who had ever experienced any type of CM were 1.74 times more likely to report SI [10].

Although the relationship between maltreatment and SI can be well supported, to effectively intervene maltreatment associated suicidal risk in children and adolescents, modifiable factors which lie in the path of this association should be further identified. Mindfulness, a concept derives from Buddhism, has been attracting considerable study interest in recent years. It refers to the practice of purposely bringing one’s attention in the present moment without judgement [11]. There are two types of mindfulness: state mindfulness, which occurs in meditation, and dispositional mindfulness (also known as trait mindfulness), which refers to a person’s predisposition to be mindful daily. Evidence has been accumulating regarding to the beneficial role of dispositional mindfulness in various mental health problems for children and adolescents, a lower level of dispositional mindfulness had been associated with higher risk of anxiety, depression, and dissociation [12, 13]. Some published studies suggested that dispositional mindfulness may play a mediating role in the association between CM and SI: a cross-sectional study of 177 young adults revealed that victims of CM reported lower level of dispositional mindfulness [14], moreover, mindfulness-based interventions were found effective in reducing SI among Chinese children and adolescents, probably through the mechanisms of reducing pain and stress, strengthening emotion regulation ability, and improving self-compassion [15–17]. Nevertheless, no existing study has ever discussed this possible mediation via mindfulness in the association between CM and SI.

In this cross-sectional study of large sample Chinese children and adolescents, we are aiming at testing the mediation of mindfulness in CM-SI association. We simultaneously investigated SI measured at three different time intervals (one-week, one-year, lifetime), compared differences in mediation proportions via mindfulness in their associations with CM.

## Methods

### Participants

In this cross-sectional study, participants were selected from two middle schools and four primary schools in Yuxi City, Yunnan Province, China, from October 14 to October 22, 2021. The selection of participants adopted a two-stage simple random cluster sampling method: in the first stage, six schools were randomly selected in Hongta District, Yuxi city; in the second stage, 3 to 4 classes were randomly selected in each chosen school, all eligible students within the chosen classes were preliminarily included. A total of 4230 students were initially identified, 3971 valid questionnaires were finally obtained. Previous studies have suggested that children cannot fully understand the concept of suicide until the age of 10 years [18], therefore only participants aged 10–17 years were further included. Other exclusion criteria include: (1) The participants themselves, their parents or legal guardians refused to participate the survey; (2) Illiterate or amentia; (3) Auditory dysfunction or language disorder; (4) Physically ill, cannot finish the survey; 5) With a past or current diagnosis of psychiatric disorders. Informed consents were obtained from the participants’ parents or legal guardians before the study. The study protocol was reviewed and approved by the Ethics Review Committee of Kunming Medical University.

### Measurements

This survey adopts a comprehensive self-administered questionnaire to collect information from the participants. The questionnaire includes a total of eight parts, this study used the following parts: basic information (such as demographics, family features, socioeconomic status, etc.), childhood maltreatment, suicidal ideation, mindfulness, depression and anxiety.

#### Childhood maltreatment

The Child Trauma Questionnaire (CTQ) used in this survey was developed in 1998 by Bernstein, the scale contains 28 questions and can be divided into 6 dimensions. In this study we used the 25 questions related to five specific types of maltreatment: emotional abuse (EA), emotional neglect (EN), physical abuse (PA), physical neglect (PN), and sexual abuse (SA). Each question was measured by using a 5-point Likert style response, indicating the frequency of a specific CM scenario [19]. The following recommended cutoffs were used to dichotomize study subjects as recommended: 8 for PA, 9 for EA, 6 for SA, 8 for PN, 10 for EN [19]. The Chinese version of CTQ provides good matching property and conception validity [20]. The Cronbach’s alphas of the CTQ based on our study sample were: 0.67 (95% CI: 0.66–0.69) for PA, 0.71 (95% CI: 0.69–0.72) for EA, 0.54 (95% CI: 0.51–0.56) for SA, 0.42 (95% CI: 0.38–0.44) for PN, 0.69 (95% CI: 0.67–0.71) for EN, and 0.67 (95% CI: 0.65–0.68) for the whole scale.

#### Suicidal ideation

During the analysis, SI was included as a binary variable (yes/no). Lifetime SI was mainly measured by using Chinese version of the Beck Scale for Suicidal Ideation (BSSI), a widely used 10-item instrument for assessing suicidality [21]. Respondents with the BSSI scores over 10 points or answered “Little”/“Sometimes”/“Often”/“Very often” to the question “During the past 12 months, have you ever seriously thought about committing suicide?” were defined as lifetime suicide ideators. One-week SI was deemed confirmative when participants answered “Weak”/“Moderate to strong” to the question “Desire to make active suicide attempt in the latest week”, or, answered “Would leave life/death to chance”/“Would avoid steps necessary to save or maintain life” to the question “Passive suicidal desire in the latest week”. The criteria for participants to be defined as one-year suicide ideators were generally comparable to that of one-week SI, only plus answered “Little”/“Sometimes”/“Often”/“Very often” to the question “During the past 12 months, have you ever seriously thought about committing suicide?”. In our study sample, the Cronbach’s alpha was 0.89 (95% CI: 0.87–0.89) for the BSSI. During the survey, for participants who reported severe SI, defined as answered “Often” or “Very often” to SI related questions, professional pediatric psychiatrist deployed at the site will perform a quick diagnosis session by using the Kiddie Schedule for Affective Disorders and Schizophrenia for School-age Children-Present and Lifetime version (K-SADS-PL), following the Diagnostic and Statistical Manual of Mental Disorders (Fifth Edition) (DSM-5). If any diagnosis of psychiatric disorders had been reached, the respondents will then be referred to local psychiatric hospitals.

#### Mindfulness

Chinese version of the Five Facet Mindfulness Questionnaire (FFMQ) has been found valid in measuring dispositional mindfulness [11]. The scale contains 39 items rated from 1 (never or very rarely true) to 5 (very often or always true). It can be divided into 5 dimensions (observing, describing, acting with awareness, nonjudging of inner experience, nonreactivity to inner experience). The total score can be summed up to 174, and a higher score is indicative of a higher level of mindfulness [22]. The Chinese FFMQ has been found acceptable internal consistency and test-retest reliability [11]. The Cronbach’s alpha for FFMQ in the current study was 0.72 (95% CI: 0.71–0.73).

#### Depression and anxiety

Depression and anxiety were included as possible confounders. They were measured by using the Patient Health Questionnaire-9 (PHQ-9) and the Generalized Anxiety Disorder-7 (GAD-7), respectively. The PHQ-9 and GAD-7 consist of 9 and 7 items on a four-point Likert-type scale ranging from 0 (Never) to 3 (almost every day). A higher combined score indicates severer symptoms of depression or anxiety [23, 24]. In this study we used an identical cut-off of 5 for the PHQ-9 and GAD-7 to dichotomize study subjects as recommended [23, 24]. The Cronbach’s alphas of the PHQ-9 and GAD-7 for our study sample were 0.88 (95% CI: 0.87–0.88) and 0.91 (95% CI: 0.90–0.91).

### Statistical analysis

The demographic characteristics and psychometric variables of the subjects were described by descriptive statistics. The chi-squared test was used to preliminarily evaluate the association between CM and SI. Univariate and multivariate binary logistic regression models were used to estimate the crude and adjusted associations between CM, mindfulness, and SI measured at three different time intervals (one-week, one-month, lifetime). In the regression analysis, CM was treated as a binary variable (yes/no), with “yes” refers to respondents who reported any type of CM based on the aforementioned cut-offs, covariates to be screened for and included into the multivariate model include demographics (age, sex, whether single child of the family, whether left-behind children, etc.), familial or socioeconomic features (marital status of the parents, family income, education levels of the parents, etc.), depression and anxiety. Path analysis was then used to test the hypothesized mediation of mindfulness in the association between CM and SI. We subsequently discussed the mediation of mindfulness for the five different types of CM also by using path analysis. All statistical analyses in this study were operated using the R software (Version 4.1.1). Adjustments were made by using R packages for survey data consistently, such as “survey” and “lavaan.survey”. The significance level for univariate logistic regression models was set as *p* < 0.1, for all the rest analyses, it was set as *p* < 0.05, two-tailed. For analyses which involve multiple comparisons, the Bonferroni’s correction will be used to adjust for significance level to avoid increased risk of inference error.

## Results

### Descriptive characteristics

Five hundred sixteen respondents were excluded based on exclusion criteria or due to missing data in the questionnaire, 3455 participants were eventually included in the analysis, with an effective response rate of 87%. Table 1 lists major characteristics of analyzed participants: the mean of age was 13.1 years; ethnic minorities accounted for 24%; girls took a larger proportion (52.3%); 73.1% respondents lived in urban areas; left-behind children, which defined as children or adolescents under 18 years old who stay at home while one of or both of the parents migrated to other localities for work, and the separation exceeded a period of six consecutive months in the past year [25], only accounted for 6.3%. After adjusted for cluster sampling design, prevalence rates and their 95% CIs for SI were: 13.0% (95% CI: 11.9–14.1%) for one-week SI, 16.3% (95% CI: 15.1–17.6%) for one-year SI, and 49.6% (95% CI: 47.9–51.2%) for lifetime SI. The prevalence for any CM was 54.5% (95% CI: 52.8–56.2%), and 18.3% (95% CI: 17.0–19.6%) for EA, 11.3% (95% CI: 10.3–12.4%) for PA, 6.5% (95% CI: 5.6–7.3%) for SA, 46.6% (95% CI: 44.9–48.3%) for EN, 25.8% (95% CI: 24.4–27.3%) for PN. The average score of mindfulness was 123, with a standard deviation of 13.9.

**Table 1:** Major characteristics of study subjects (*N* = 3455)

Characteristics	*N* (%)	Mean ± SD
Gender		
Boys	1647 (47.7)	
Girls	1808 (52.3)	
Ethnicity		
Han majority	2625 (76)	
Minorities	830 (24)	
Age (Years)		13.1 (2.25)
Residence		
Township	2525 (73.1)	
Village	930 (26.9)	
Study type		
Day students	2205 (63.8)	
Boarding students	1250 (36.2)	
Single child: Yes	1562 (45.2)	
Grade		
Primary school	731 (21.2)	
Junior high school	1373 (39.7)	
Senior high school	1351 (39.1)	
Left-behind children: Yes	218 (6.3)	
Educational level of father		
Elementary school and below	386 (11.2)	
Junior high school and above	3069 (88.8)	
Educational level of mother		
Elementary school and below	349 (10.1)	
Junior high school and above	3106 (89.9)	
Marital status of the parents		
In marriage	3117 (90.3)	
Divorced/Re-married/Widowed	338 (9.7)	
Family income		
Stable	3410 (98.7)	
Unstable	45 (1.3)	
Mindfulness score (FFMQ)		123 (13.9)
SI		
One-week SI	449 (13)	
One-year SI	564 (16.3)	
Lifetime SI	1712 (49.6)	
Childhood maltreatment		
Any	1883 (54.5)	
Emotional abuse (EA)	633 (18.3)	
Physical abuse (PA)	391 (11.3)	
Sexual abuse (SA)	223 (6.5)	
Emotional neglect (EN)	1610 (46.6)	
Physical neglect (PN)	893 (25.8)	
Depression: Yes (PHQ-9 score ≥ 5)	1310 (37.9)	
Anxiety: Yes (GAD-7score ≥ 5)	818 (23.7)	

*CTQ* Childhood Trauma Questionnaire; *SI* Suicidal ideation; *FFMQ* Five Facet Mindfulness Questionnaire; *PHQ-9* Patient Health Questionnaire-9; *GAD-7* Generalized Anxiety Disorder-7

### Associations between CM, SI, and mindfulness

Chi-squared test showed that, CM involvement was significantly associated with all SI indicators (Table 2). Moreover, compared with one-week and one-year SI, lifetime SI was the most frequently reported among adolescents who had experienced CM, with a prevalence of 62.6% (95% CI: 69.3–75.3%). By using univariate and multivariate logistic regression models, we found that CM experience was in general significantly associated with increased odds of SI: with adjusted ORs of 2.95 (95% CI: 2.22–3.96), 2.71 (95% CI: 2.35–3.97), and 2.31 (95% CI: 1.82–2.51) for one-week, one-year, and lifetime SI. Meanwhile, mindfulness was negatively associated with SI: every 5 points increase in the FFMQ score was associated with a similar 2% reduction in odds for all three SI indicators (Table 3). We also analyzed the association between CM and mindfulness by using multivariate linear model, and the results indicated a significant inverse association between the two variables (*b* = − 5.12, *p* < 0.001) (see in supplementary materials, Table S1).

**Table 2:** Associations between childhood maltreatment and SI indicators

\SI indicators	Childhood maltreatment (*N*, %)		*χ*^*2*^ statistic	*p* value
	Yes	No		
One-week SI				
Yes	381 (20.2)	68 (4.3)	191.75	< 0.001
No	1502 (79.8)	1504 (95.7)		
One-year SI				
Yes	470 (25.0)	94 (6.0)	225.96	< 0.001
No	1413 (75.0)	1478 (94.0)		
Lifetime SI				
Yes	1179 (62.6)	533 (33.9)	282.44	< 0.001
No	704 (37.4)	1039 (66.1)		
Total	1883	1572	–	–

**Table 3:** Univariate and multivariate logistic regression models fitting results for associated factors of SI indicators

Variables	One-week SI		One-year SI		Lifetime SI	
	Univariate	Multivariate	Univariate	Multivariate	Univariate	Multivariate
	OR (90% CI)	OR (95% CI)	OR (90% CI)	OR (95% CI)	OR (90% CI)	OR (95% CI)
Anxiety: GAD-7 score ≥ 5 (Ref: GAD-7 < 5)		3.21 (2.41, 4.29)		3.05 (2.35, 3.97)		2.33 (1.83, 2.96)
Depression: PHQ-9 score ≥ 5 (Ref: PHQ-9 < 5)		2.71 (1.98, 3.73)		4.60 (3.43, 6.20)		2.81 (2.30, 3.45)
Mindfulness score: + 5 points	0.95 (0.94, 0.96)	0.98 (0.97, 0.99)	0.95 (0.94, 0.95)	0.98 (0.97, 0.99)	0.95 (0.94, 0.95)	0.97 (0.97, 0.98)
Childhood maltreatment: Yes (Ref: No)	5.61 (4.50, 7.06)	2.95 (2.22, 3.96)	5.23 (4.31, 6.38)	2.71 (2.35, 3.97)	3.26 (2.90, 3.67)	2.13 (1.82, 2.51)
Gender: Girls (Ref: Boys)	1.71 (1.44, 2.03)	1.42 (1.12, 1.79)	2.16 (1.84, 2.53)	1.88 (1.51, 2.37)	1.84 (1.65, 2.07)	1.81 (1.55, 2.12)
Age: + 1 year	0.92 (0.89, 0.96)	1.02 (0.90, 1.16)	0.98 (0.95, 1.02)		1.09 (1.06, 1.12)	1.03 (0.95, 1.13)
Ethnicity: Minorities (Ref: Han majority)	1.16 (0.96, 1.40)		1.01 (0.84, 1.19)		1.05 (0.92, 1.20)	
Residence: Village (Ref: Township)	0.83 (0.68, 1.01)		0.62 (0.51, 0.75)	0.63 (0.46, 0.86)	0.87 (0.77, 0.99)	0.66 (0.53, 0.82)
Study style: Boarding students (Ref: Day students)	0.55 (0.46, 0.66)	1.01 (0.54, 1.90)	0.65 (0.55, 0.77)	1.46 (0.82, 2.67)	1.13 (1.01, 1.26)	0.98 (0.67, 1.45)
Single child: No (Ref: Yes)	1.18 (0.99, 1.40)		0.94 (0.81, 1.10)		1.07 (0.95, 1.19)	
Educational level of father: Junior high school and above (Ref: Elementary school and below)	1.09 (0.84, 1.44)		1.26 (0.98, 1.63)		1.08 (0.90, 1.29)	1.27 (0.97, 1.66)
Educational level of mother: Junior high school and above (Ref: Elementary school and below)	0.82 (0.63, 1.07)		0.93 (0.73, 1.21)		0.74 (0.62, 0.90)	0.75 (0.57, 0.98)
Family income: Unstable (Ref: Stable)	2.47 (1.37, 4.25)	1.57 (0.70, 3.32)	2.61 (1.51, 4.36)	1.98 (0.90, 4.18)	2.53 (1.50, 4.47)	1.89 (0.92, 4.09)
Left-behind children: No (Ref: Yes)	0.46 (0.35, 0.61)	0.60 (0.41, 0.89)	0.50 (0.38, 0.65)	0.68 (0.46, 1.01)	0.60 (0.47, 0.75)	0.76 (0.55, 1.06)
Marital status of the parents: Divorced/Re-married/Widowed (Ref: In marriage)	1.77 (1.38, 2.25)	1.04 (0.74, 1.45)	2.25 (1.81, 2.78)	1.38 (1.00, 1.90)	1.72 (1.42, 2.08)	1.14 (0.87, 1.49)
Grade (Ref: Primary school)						
Junior high school	1.14 (0.92, 1.41)	0.62 (0.39, 0.98)	1.88 (1.53, 2.33)	0.92 (0.58, 1.44)	1.93 (1.65, 2.25)	1.31 (0.96, 1.80)
Senior high school	0.56 (0.44, 0.71)	0.19 (0.07, 0.48)	0.95 (0.76, 1.19)	0.21 (0.08, 0.52)	1.78 (1.52, 2.08)	0.84 (0.44, 1.60)

### Mediation of mindfulness in CM-SI association

**Fig. 1:**
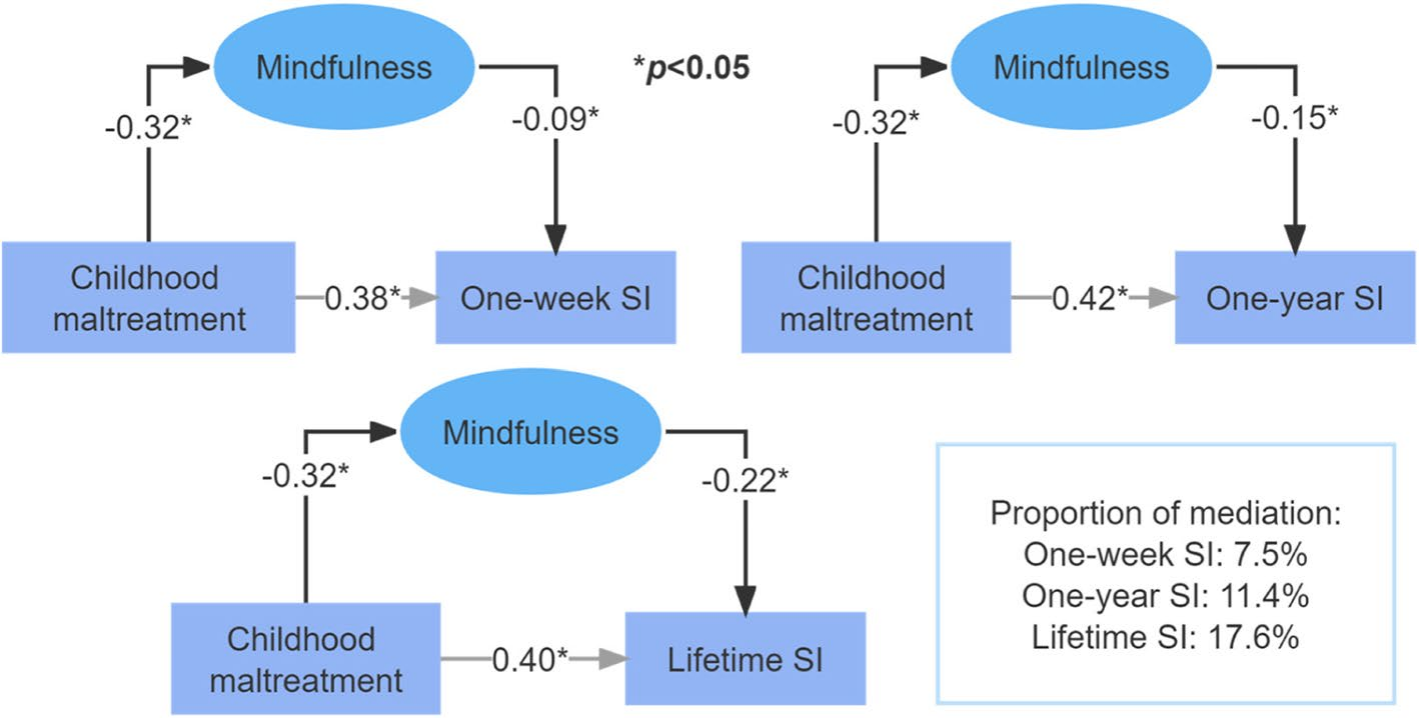
The hypothetical path model of childhood maltreatment, mindfulness and three types of SI

**Fig. 2:**
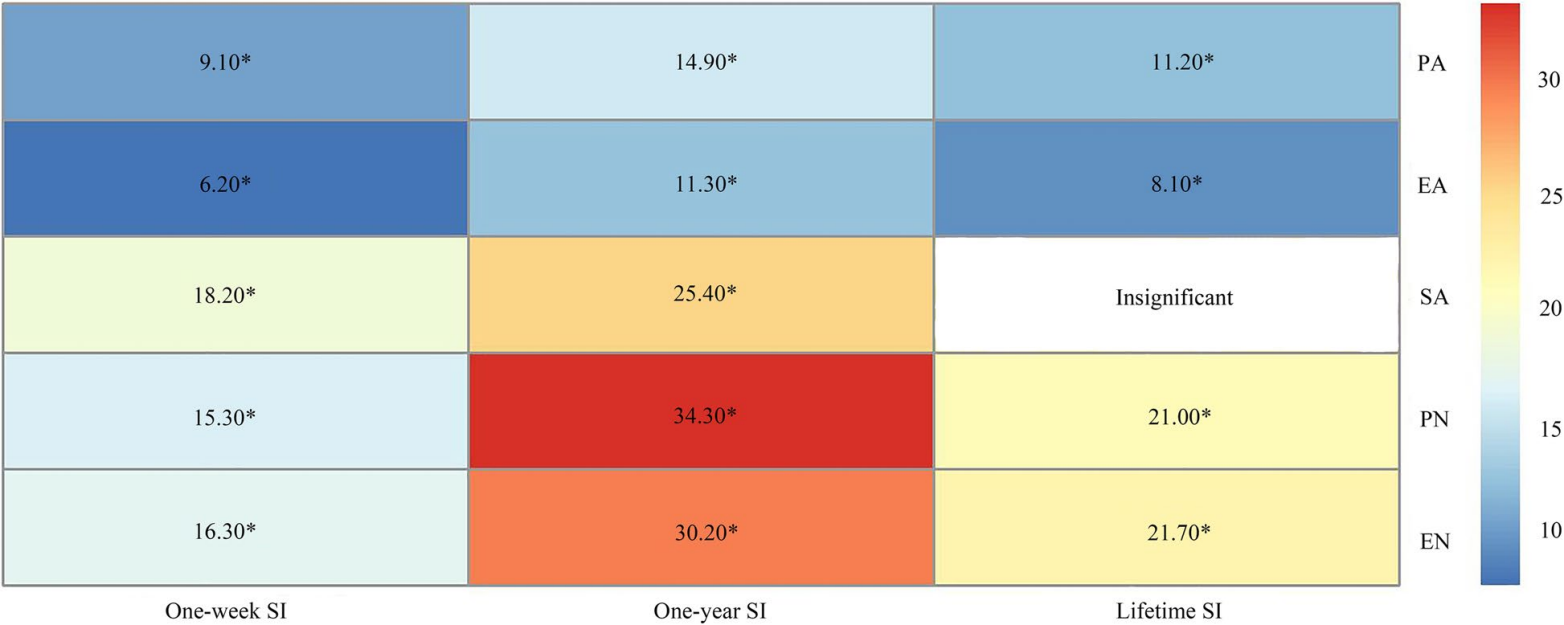
Proportions (%) of mediation by mindfulness in the associations between different types of childhood maltreatment and SI indicators. PA: Physical abuse; EA: Emotional abuse; SA: Sexual abuse; EN: Emotional neglect; PN: Physical neglect. (^*^p < 0.05 based on Bonferroni’s correction)

Results from multivariate analyses suggested a possible mediation of mindfulness in the association between CM and SI, therefore three hypothetical path models had been constructed for three SI indicators separately: mindfulness presented as a significant mediator, accounted for 7.5, 11.4, and 17.6% of the total associations for one-week SI, one-year SI, and lifetime SI (Fig. 1). We further constructed a series of path models to evaluate the mediation of mindfulness for the associations between specific types of CM and SI indicators, and the results were jointly summarized in Fig. 2: mindfulness significantly mediated their associations with SI indicators for all types of CM, and the proportion of mediation was the highest for PN and one-year SI (34.3%), followed by EN and one-year SI (30.2%), SA and one-year SI (25.4%).

## Discussion

In this cross-sectional study with a large sample of 3455 Chinese children and adolescents, we found that CM was significantly associated with increased odds of SI. Besides, a significant mediation via mindfulness has been revealed in this association: mindfulness not only prominently mediated the association between CM involvement and SI in general, but also presented as noticeable mediator in their associations with SI indicators for all types of CM. These major findings of the current study highlight the promising role of mindfulness in preventing suicidal risk for childhood maltreatment victims.

Compared to adolescents who had not experienced CM, victims are seen statistically higher odds of SI. This finding was in agreement with two previous large-scale population-based longitudinal studies [9, 10]. The connection between CM and suicidality has been repeatedly validated. On one hand, people who had experienced CM are usually overwhelmed to respond to or handle emotions, or observed higher propensity of psychopathology (such as interpersonal sensitivity, depression, anxiety, etc.) [26], these factors are all direct or indirect relate to SI [3, 4]. On the other hand, CM victims tend to develop social isolation and report reduced social support, both of them are associated with increased risk of suicide [27, 28]. Moreover, suicide interpersonal theory suggests that people who had experienced negative events, such as CM, may develop a pain-adapted feeling, have a diminished fear of self-harm, and eventually lead to increased suicide related behaviors [29].

As expected, we did find a significant mediation via mindfulness in the association between CM and SI. This mediation can also be well justified by existing literature: for instance, some studies reported that a lower level of mindfulness was associated with symptoms of depression and anxiety, or higher risk of non-suicidal self-injury [12, 13], all recognized risk factors of suicide. Besides, interventional studies suggested that, through training or interventions to improve mindfulness, a significant decrease in SI had been observed [15]. As to the relationship between CM and mindfulness, it has been reported that people who suffered from CM were observed lower level of mindfulness [12, 14]. Moreover, CM was negatively correlated with self-compassion [16], and it has been found that mindfulness-based interventions can improve self-compassion [17].

Although significant mediation via mindfulness has been revealed for all types of CM and SI indicators, the proportion of mediation was particularly high for PN, EN, and SA, accounted for 34.3, 30.2, and 25.4% of the total associations. Compared with abuse, the physical and psychological damage of neglect is usually less severe. For instance, abuse was related to higher risk of depression and non-suicidal self-injury than neglect [7, 30]. Some studies provide direct support on a stronger association between abuse and SI [31, 32]. It has been suggested that interpersonal interaction intense intervention measures (e. g. daily group treatment) may lead to severe distress in patients who had experienced emotional abuse due to maladaptive interpersonal schemas [33]. Meanwhile, in a newly published study, the authors stated that for borderline personality disorder patients who reported childhood neglect experience, after short-term intensive dialectical behavior therapy, their depressive symptoms had been substantially reduced; nevertheless, for patients of childhood abuse, a higher withdrawal rate and poorer intervention effect were observed [34]. Therefore, it is reasonable to suspect that, the mental health of people who had experienced childhood neglect other than abuse can be more effectively intervened.

Another interesting finding of the current study is that, among all types of abuse, we found a stronger mediation by mindfulness in its associations with one-week SI and one-year SI for SA. Compared with PA or EA, SA imposes even greater physical and psychological damage to the victims [35, 36]. Research evidence also indicates that, of the five types of CM, SA showed the strongest association with SI [31, 37]. In regard of this, this finding of our study may suggest that, although SA victims suffer from more devastating mental health impairment, mindfulness based intervention measures could still be effective in reducing their suicidal risk [38, 39].

The major findings of our study probably suggest that mindfulness based intervention measures could be effective in preventing maltreatment associated suicide for children and adolescents. Although mindfulness to some extant is an inherent state [40], it can be effectively improved by uncomplicated intervention methods, such as meditation practices [41], pressure relief training [42], yoga [40], etc. In several large-scale randomized controlled trials of adolescents and children from Western countries, compared with controls, participants who accepted school-based mindfulness interventions, such as mindfulness-based stress reduction, daily mindfulness meditation practice, showed significant improvement in negative emotions, coping strategies, health-related quality of life, and SI [41–43]. In China, although some mindfulness intervention studies had already been done with positive conclusion, nearly all of them were based on small sample size of children and adolescents recruited at clinical setting [44, 45]. Therefore, the effectiveness of mindfulness based intervention programs in the general Chinese children and adolescent population should be further investigated.

The main novelty of the current study lies in the fact that it is among the first attempts to elaborately explore the mediation of mindfulness in the association between CM and SI among general Chinese children and adolescents. Population-based sampling design further consolidates the validity of study results. Even though, several limitations should be pointed out: first, the nature of our study is cross-sectional, therefore causal inferences cannot be made; second, the Cronbach’s alphas for the instrument measuring different types of CM were generally low, particularly for PN and SA, therefore the risk of information bias should be noticed; third, as the major aim of our study was focusing on investigating the mediation of mindfulness in CM-SI association, we only adopted a very loose criterion to define SI, in order to include more positive cases into the subsequent analysis to guarantee higher statistical power, therefore the prevalence rates that we calculated are much higher than previously published studies for Chinese children and adolescents, which used different instruments and generally more strict standard to define SI. Finally, the study sample was drawn from a locality within Yunnan province, the generalization of our study results to the general Chinese children and adolescent population should be cautiously made.

## Conclusions

To conclude, in this population-based cross-sectional study of 3455 Chinese children and adolescents, we detected a prominent mediation of mindfulness in the association between CM and SI. More importantly, this mediation varied for different types of CM, and was the strongest for PN, EN, and SA. Our study findings highlight the intervention potential of mindfulness in preventing CM associated suicidal risk in Chinese children and adolescents. Prospective studies of large and more representative samples are warranted to further validate our major results.

## Supplementary Information

**Additional file 1.**

## Data Availability

The analytical database of this study can be obtained from the corresponding author under reasonable request.
