# Supplementary material for: Childhood maltreatment and suicidal ideation in Chinese children and adolescents: the mediating role of mindfulness
Source: BMC Psychiatry. 2022 Nov 4;22:680. doi: 10.1186/s12888-022-04336-w (PMC9635069; doi:10.1186/s12888-022-04336-w)
Supplement: Supplementary file 1 — Additional file 1. [file 12888_2022_4336_MOESM1_ESM.docx]

| **Table S1** Associated factors of mindfulness by univariate and multivariate linear regression models | | | | |
| --- | --- | --- | --- | --- |
| Covariates | Mindfulness score | | | |
|  | Univariate | | Multivariate | |
|  | *b* | *p* | *b* | *p* |
| Anxiety: Yes (GAD-7 score ≥ 5) (Ref: GAD-7 score < 5) |  |  | -5.21 | <0.01 |
| Depression: Yes (PHQ-9 score ≥ 5) (Ref: PHQ-9 score < 5) |  |  | -5.59 | <0.01 |
| Childhood maltreatment: Yes (Ref: No) | -8.19 | <0.01 | -4.37 | <0.01 |
| Gender: Female (Ref: Male) | -1.15 | 0.02 | 1.07 | 0.01 |
| Age: +1 year | -1.11 | <0.01 | 0.14 | 0.53 |
| Ethnicity: Minorities (Ref: Han majority) | -0.28 | 0.62 |  |  |
| Residence: Village (Ref: Township) | -5.06 | <0.01 | -3.45 | <0.01 |
| Study type: Boarding students (Ref: Day students) | -4.66 | <0.01 | -1.65 | 0.11 |
| Single child: No (Ref: Yes) | -2.86 | <0.01 | -1.15 | 0.01 |
| Educational level of father: Junior high school and above  (Ref: Elementary school and below) | 4.79 | <0.01 | 2.18 | <0.01 |
| Educational level of mother: Junior high school and above  (Ref: Elementary school and below) | 5.14 | <0.01 | 1.15 | 0.11 |
| Family income: Unstable (Ref: Stable) | -4.85 | 0.02 | -0.77 | 0.66 |
| Left-behind children: No (Ref: Yes) | 4.41 | <0.01 | 1.42 | 0.09 |
| Marital status of the parents: Divorced/Re-married/Widowed  (Ref: In marriage) | -4.38 | <0.01 | -2.39 | <0.01 |
| Grade (Ref: Primary school) |  |  |  |  |
| Junior high school | -3.14 | <0.01 | -2.56 | <0.01 |
| Senior high school | -6.46 | <0.01 | -1.74 | 0.30 |
